# Human Ovarian Follicular Fluid Mesenchymal Stem Cells Express Osteogenic Markers When Cultured on Bioglass 58S-Coated Titanium Scaffolds

**DOI:** 10.3390/ma16103676

**Published:** 2023-05-11

**Authors:** Federica Riva, Nora Bloise, Claudia Omes, Gabriele Ceccarelli, Lorenzo Fassina, Rossella Elena Nappi, Livia Visai

**Affiliations:** 1 Histology and Embryology Unit, Department of Public Health, Experimental and Forensic Medicine, University of Pavia, 27100 Pavia, Italy; federica.riva01@unipv.it; 2 Department of Molecular Medicine, Centre for Health Technologies (CHT), INSTM UdR of Pavia, University of Pavia, 27100 Pavia, Italy; nora.bloise@unipv.it; 3 Medicina Clinica-Specialistica, UOR5 Laboratorio di Nanotecnologie, ICS Maugeri, IRCCS, 27100 Pavia, Italy; 4 Center for Reproductive Medicine, Obstetrics and Gynecology Unit 2, Woman and Child Health Department, Fondazione IRCCS Policlinico San Matteo, 27100 Pavia, Italy; r.nappi@unipv.it; 5 Human Anatomy Unit, Department of Public Health, Experimental and Forensic Medicine, Centre for Health Technologies (CHT), University of Pavia, 27100 Pavia, Italy; gabriele.ceccarelli@unipv.it; 6 Department of Electrical, Computer and Biomedical Engineering, Centre for Health Technologies (CHT), University of Pavia, 27100 Pavia, Italy; lorenzo.fassina@unipv.it; 7 Department of Clinical, Surgical, Diagnostic and Pediatric Sciences, University of Pavia, 27100 Pavia, Italy

**Keywords:** human ovarian follicular fluid (hFF) mesenchymal stem cells (MSCs), bone tissue engineering, biomaterials, bioglass–titanium, osteogenic differentiation

## Abstract

Recent studies have reported that stem cells (human follicular fluid mesenchymal stem cells or hFF-MSCs) are present in ovarian follicular fluid (hFF) and that they have a proliferative and differentiative potential which is similar to that of MSCs derived from other adult tissue. These mesenchymal stem cells, isolated from human follicular fluid waste matter discarded after retrieval of oocytes during the IVF process, constitute another, as yet unutilized, source of stem cell materials. There has been little work on the compatibility of these hFF-MSCs with scaffolds useful for bone tissue engineering applications and the aim of this study was to evaluate the osteogenic capacity of hFF-MSCs seeded on bioglass 58S-coated titanium and to provide an assessment of their suitability for bone tissue engineering purposes. Following a chemical and morphological characterization with scanning electron microscopy (SEM) and energy dispersive spectroscopy (EDS), cell viability, morphology and expression of specific osteogenic markers were examined after 7 and 21 days of culture. The hFF-MSCs seeded on bioglass and cultured with osteogenic factors, when compared with those seeded on tissue culture plate or on uncoated titanium, exhibited enhanced cell viability and osteogenic differentiation, as reflected by increased calcium deposition and increased ALP activity with expression and production of bone-related proteins. Taken together, these results demonstrate that MSCs from human follicular fluid waste materials can be easily cultured in titanium scaffolds coated with bioglass, having osteoinductive properties. This process has significant potential for regenerative medicine applications and indicates that hFF-MSCs may be a valid alternative to hBM-MSC cells in experimental models in bone tissue engineering.

## 1. Introduction

Organ and tissue transplantation, whether donor-derived or artificial [1], is a central and established form of treatment but it is not without significant challenges: donor availability is limited and immunological rejection or diseases associated with transplantation are not uncommon. Tissue engineering is a promising approach to counter these challenges and significant recent advances in tissue engineering mean that we are now able to construct bio-artificial tissues of an appropriate shape and size to repair damaged tissues [2].

Bone tissue (BT), the context of this paper, is composed of an extracellular matrix which is calcified and rich in inorganic and organic compounds. The histogenesis of BT is strongly influenced by physical stresses, including gravity and torsion, and by the physical environment more generally: the impacts of tension, compression, stretch shear stress and magnetic fields all modulate cell activity, growth and tissue remodelling [3]. Recent advances in tissue engineering and development have also allowed for the formation in vitro of bone-like artificial equivalents [4] and new tissue engineering strategies use living cells (and/or their products, such as growth factors, bioactive molecules, etc.) and innovative scaffolds to develop tissue substitutes as an alternative to inert materials. Cells, obviously, react to the environment in a consistent manner and an effective biomaterial scaffold will support cell adhesion and proliferation and the correct level of cell differentiation. The scaffolds normally used for bone regeneration combine physical supports (such as polymers, ceramics or metals) with stem cells and growth factors and, in turn, lead to the formation of new bone through stimulating natural tissue regeneration abilities [5,6]. These classes of materials can be combined into composite biomaterials for bone tissue engineering. This is the case with composite scaffolds based on natural polymers (such as alginate) and ceramics, which generally present superior levels of biocompatibility and bioactivity, best mimicking the natural functions of bone [7].

The focus of this paper is to propose an alternative method of combining materials for bone tissue engineering: we have combined a bulk structure made of biocompatible pure titanium [8] with a bioglass coating. Bioactive glass (bioglass) has been widely used as a coating for biomedical implants It supports osseointegration, antibacterial behaviour, bone formation and tissue healing by the release of ions [9,10]. As such, bioglass can function as an effective coating for titanium materials (already in use in this context): it aids biocompatibility and osteoinductivity and accelerates bone cell differentiation [11]. There have been a number of studies, assessing the regenerative potential of bioglass coatings, of the differentiation of particular kinds of stem cells into bone phenotypes. As has been documented, a number of stem cell types are particularly adaptable to bone tissue engineering. These include mesenchymal stromal stem cells (MSCs) derived from bone marrow, dental pulp, adipose tissue, umbilical cord blood, amniotic fluid, foetal membrane and placental tissues [12]. Mesenchymal progenitors may in fact also be present, as has been shown, in the tunica albuginea of the adult human ovary, as a result of an epithelial–mesenchymal transition and sequential differentiation in the components of new primary follicles, i.e., granulosa cells or even germ cells [13,14,15]. The discovery of follicular renewal and the presence of germ-line stem cells within the post-natal ovaries of mammals calls into question the established position that mammalian females do not possess the capacity to renew germ cells during the foetal period.

Graafian follicles, derived from the maturation process of the primary ovarian follicle, consist of three components: the follicular fluid, a single mature oocyte and a few thousand granulosa cells (GC). The follicular fluid is the liquid contained in the mature ovarian follicle, which is partially expelled, under normal physiological conditions, with the oocyte at the time of ovulation. During the course of the in vitro fertilization (IVF) procedure, this biological liquid is collected and screened to isolate the oocyte. The liquid itself, following the incubation of the oocyte, is discarded as waste material. This ovarian follicular liquid, however, and as we have demonstrated elsewhere in minimal cell culture conditions, contains a heterogeneous cell population which includes mesenchymal stem cells, epithelial cells and neural-like cells, all of which have been well characterized in terms of morphology and differentiation capacity [15,16]. Other studies have demonstrated the presence of epithelial stem cells which are able to differentiate into oocyte-like cells and into hepatocytes [17] and also that, interestingly, oocyte-like cells can also be differentiated by mesenchymal stem cells derived from human ovarian follicular fluid after induction with bone morphogenic protein 15 [18]. Our previous data demonstrated that the ovarian-follicular-fluid-derived mesenchymal stem cells contained in ovarian follicular waste fluid expressed the main mesenchymal stemness markers (CD44, CD73, CD90, CD105, CD146 and STRO-1) and can be induced to osteogenic, chondrogenic and adipogenic differentiation [15,16].

This indicates that hFF-MSCs could be a viable alternative source of MSCs: they derive directly from a biological waste liquid which is routinely collected during IVF procedures, and which could be used for MSC isolation. This would have no impact on oocyte pick-up. In this sense, ovarian follicular fluid could be considered an important new source of MSCs for use in experimental cell models for regenerative medicine studies and cell-based therapeutic applications [15,19,20]. Within the context of the most recent research on new sources of MSCs and on biomaterials for tissue engineering, the purpose of this study is to: (i) increase our understanding of the morphological and biochemical characterization of mesenchymal multipotent cells with stem peculiarities derived from human ovarian follicular fluid and (ii) analyse the suitability of titanium scaffolds coated with bioglass and seeded with FF-MSCs as bio-complexes for promoting bone tissue regeneration.

## 2. Materials and Methods

### 2.1. Titanium and Bioglass Coating Preparation and Characterization

Titanium disks (diameter 10 mm, thickness 0.5 mm) were purchased from Goodfellow© (Huntingdon, UK) (titanium purity 99.6%). The bioglass SiO_2_ (58 wt%)–CaO (33 wt%)–P_2_O_5_ (9 wt%) (that is, bioglass 58S) was synthesized by means of a sol–gel route [21]. An amount of 133.2 mL of TEOS (98 wt%, Sigma-Aldrich, St. Louis, MO, USA) was added to 42 mL of an aqueous solution of 0.01 N HCl and mixed with 180.4 mL of ethanol. The mixture was stirred for about 15 min until the hydrolysis was almost complete. At that point, 18.4 g of triethyl phosphate was added and the solution was stirred for 20 min. Finally, 112 g of Ca(NO_3_)_2_4H_2_O (Sigma-Aldrich) was dissolved in the solution during 20 min of magnetic stirring. To increase the number of OH^−^ groups on the Ti surface for the bioglass coating, Ti disks were activated by immersion in 0.25% NH_4_OH for 20 min and then washed with distilled water and ethanol. Ti scaffolds were then positioned on a spin coater (Speedline Technologies, model P6700 series) and 4 ÷ 5 drops of formed sol were deposited on each Ti scaffold. The spin coater was set up to reach 2000 rpm in 5 s (ramp up to 400 rpm/s) and maintained this speed for 60 s. The whole procedure was repeated 10 times for each scaffold to create a multi-layer coating. After 24 h of gelation, scaffolds were dried at 65 °C for 24 h and then at 90 °C for another 24 h. Finally, they were placed in an oven for the stabilization treatment: heating with 0.3 °C/min up to 700 °C, isotherm for 3 h at 700 °C, and then cooling to room temperature. The flowing atmosphere was N_2_ (0.3 L/min) and O_2_ (0.3 L/min) [11]. The bioglass coating thickness was measured at around 25 µm using a KLA Tencor Stylus P6 profilometer (Milpitas, CA, USA), while the Ti and bioglass-Ti scaffold surfaces were observed by scanning electron microscopy (SEM) using a Zeiss EVO-MA10 (Carl Zeiss, Oberkochen, Germany). An energy dispersive X-ray spectroscopy (EDX) detector (X-max 50 mm^2^, Oxford Instruments, Oxford, UK) was used, coupled with SEM, to obtain the elemental maps of the atomic element of both titanium and Ti-bioglass. The acceleration voltage used was 20 kV. The samples were not gold sputtered prior to EDX analysis.

### 2.2. Multipotent Follicular Mesenchymal Stem Cells (FF-MSC) Culture

Human follicular fluids were collected as residual samples during the assisted reproductive techniques of oocyte collection from female ovaries by transvaginal ultrasound-guided aspiration using a single aspiration needle (Cook Medical, Bloomington, IN, USA) and phenotypically analysed to assess their mesenchymal properties (Figure S1) [15]. Mesenchymal stem cells present in the hFF were isolated by centrifuging the follicular fluid by density gradient separation (Lymphoprep, NycomedPharma, Oslo, Norway) for 30 min at 1800 rpm (to eliminate red blood cells and debris), as reported in [15]. hFF-MSCs were cultured at 37 °C in a humidified incubator with 5% CO_2_ in DMEM medium (Sigma-Aldrich) supplemented with 10% foetal bovine serum (FBS, Eurobio, Les Ulis, France), 2 mM of L-glutamine (Eurobio), 100 mg/mL of penicillin (Eurobio) and 100 μg/mL of streptomycin (Eurobio). Cells were used for all these experiments at the first passage of cell culture.

### 2.3. Immunostaining and Fluorescence Microscopy Analysis

After isolation and after 21 days of culture on the different scaffolds at 37 °C in a humidified incubator with 5% CO_2_, hFF-MSCs were fixed with 4% paraformaldehyde for 1 h at room temperature, washed two times in PBS, and incubated for 20 min with PTA blocking solution (1% BSA and 0.02% Tween 20 in PBS). The samples were subsequently incubated for 1 h at room temperature with mouse primary antibody monoclonal anti-vimentin (Sigma-Aldrich), diluted 1:100 in PTA. After three washes with PTA, the samples were incubated respectively with anti-mouse FITC-conjugated antibody, diluted 1:100 in PTA (Sigma-Aldrich) for 30 min at room temperature. Then, the cells were washed in PBS and nuclear DNA was counterstained with 0.5 μg/mL Hoechst 33258 (Sigma-Aldrich). Finally, the cells were observed by a TCS SPII confocal microscope (Leica Microsystems, Bensheim, Germany) equipped with a digital image capture system at 20× magnification and images analysis was performed by ImageJ software (https://imagej.nih.gov/ij/, accessed on 8 February 2023) to quantify the fluorescence level inside the cells (Figure S2).

### 2.4. Experimental Design

On day 0, a drop of hFF-MSCs containing about 5 × 10^4^ cells was added to the top of sterile titanium and Ti-bioglass scaffolds (diameter 10 mm) or on the control (plastic tissue culture plate or glass coverslip) and, after 0.5 h, 0.5 mL of culture medium was added to the scaffolds. Cell adhesion was evaluated in terms of focal adhesion activation after 24 h from seeding and cell viability was evaluated at different time points up to 21 days of culture. Osteogenic differentiation was induced after 1 week of hFF-MSC culture using DMEM medium supplemented with 10% FBS, 0.1 mM ascorbic acid 2-phosphate, 10^−2^ M β-glycerol phosphate and 10^−8^ M dexamethasone [22] for 21 days, changing the medium every 2 days. After 3 weeks, the samples were processed to test cell viability, and for molecular and biochemical analysis.

### 2.5. Analysis by Confocal Laser Scanning Microscope (CLSM)

For focal adhesion detection, 24 h from seeding, cell-seeded scaffolds and the control were fixed with 4% (*w*/*v*) paraformaldehyde solution in a 0.1 M phosphate buffer (PBS) (pH 7.4) for 30 min at 4 °C and washed with PBS three times for 15 min. The scaffolds were then incubated with PTA (PBS containing 0.02% (*v*/*v*) Tween 20 and 1% (*w*/*v*) BSA) for 2 h at room temperature and washed. Anti-p-FAK antibody (pY397, 1:250, Santa Cruz Biotechnologies, Santa Cruz, CA, USA) was used as the primary antibody and incubated overnight at 4 °C. Negative controls were incubated directly in PTA overnight at 4 °C, without the primary antibody. The scaffolds, controls and negative controls were washed and incubated with Alexa Fluor 488 goat anti-rabbit IgG (molecular probes) at a dilution of 1:500 in PTA for 1 h at room temperature. At the end of the incubation, the scaffolds and controls were washed in PBS, counterstained with a solution 0.5 μg/mL Hoechst 33258 (Sigma-Aldrich) to target the cellular nuclei, and then washed with PBS. The images were taken by the TCS SPII confocal microscope (Leica Microsystems) equipped with a digital image capture system at 40× and 60× magnification, acquiring images every 1.5 μm to 100 μm of depth. The fluorescence background of the negative controls was almost negligible.

### 2.6. Western Blot Analysis

hFF-MSCs cultured for 24 h onto TCPS (control), titanium and Ti-bioglass disks were lysed with RIPA buffer (50 mM Tris-HCl, pH 7.6, 150 mM NaCl, 0.25% sodium deoxycholate, 0.1% SDS, 1 mM EDTA and protease inhibitors, Sigma-Aldrich) and centrifuged (12,000 g, 10 min, and 4 °C). The total proteins of supernatants were determined by the bicinchoninic acid assay (BCA Protein Assay; Pierce, Waltham, MA, USA) and equal amounts were size-fractionated in SDS-PAGE under reducing conditions, transferred to nitrocellulose membranes (200 mA; 4 °C, 3 h) and stained with red Ponceau. Membranes were blocked in BSA 3% in TBS-T buffer (0.9% NaCl, 0.02 M Tris pH 7.5, and 0.05% Tween-20; 1 h; RT) before incubation with the following anti-human primary antibodies for cell adhesion: mouse monoclonal anti-β-actin (1:500), rabbit polyclonal anti-focal adhesion kinase (FAK; 1:100) and rabbit polyclonal anti-anti-phosphorylated FAK (pTyr397-specific; 1:100) from Santa Cruz Biotechnologies. Detection was performed with appropriate peroxidase-labelled secondary antibodies in TBS-T buffer and an enhanced chemiluminescence (ECL) kit (Amersham, GE Healthcare, Waukesha, WI, USA). To capture images of the bands, an ImageQuant™ LAS 4000 mini-biomolecular imager (GE Healthcare) was used, and the images were quantified with ImageJ software.

### 2.7. Cell Viability Assay

To assess the viability of hFF-MSCs cultured on control, titanium and Ti-bioglass disks the mitochondrial activity was evaluated using a resazurin-based assay (TOX8-1KT, Sigma-Aldrich) after 1, 7 and 21 days of incubation. According to the manufacturer’s instructions, the culture medium was replaced by 10% (*v*/*v*) resazurin solution in a fresh culture medium, incubating all samples for 3 h at 37 °C in 5% CO_2_. At the end of incubation time, the absorbance of the samples was measured at a wavelength of 600 nm with a reference wavelength of 690 nm using a microplate reader (Bio-Rad Laboratories, Hercules, CA, USA). A standard curve of cell viability was used to obtain the cell number per sample.

### 2.8. Scanning Electron Microscopy (SEM) Analysis

hFF-MSCs morphological observations were performed after 21 days of culture. Cells cultivated on titanium, Ti-bioglass and on thermanox disks (as a control) were fixed with a 2.5% (*v*/*v*) glutaraldehyde solution in 0.1 M Na-cacodylate buffer (pH 7.2) for 1 h at 4 °C, washed with Na-cacodylate buffer and then dehydrated at room temperature in gradient ethanol series up to 100% [23]. To obtain a complete state of dehydration, the samples were kept in 100% ethanol for 15 min, and then critical-point-dried with CO_2_ to lyophilize for 24 h. The samples were then sputtered with gold particles, to increase their conductivity and obtain better images, and then observed at 3000× and 5000× magnification using a Zeiss EVO-MA10 SEM (Carl Zeiss).

### 2.9. Alkaline Phosphatase (ALP) Assay

To study the osteoblast differentiation of hFF-MSCs after 21 days of culture in a culture medium with or without osteogenic factors, ALP content was determined using a colorimetric end point assay [24,25]. This method measures the colourless conversion of the p-nitrophenol phosphate (pNPP) substrate hydrolysed by the ALP enzyme to the yellow p-nitrophenol product, where the colour change rate corresponds to the amount of enzyme in the solution. Briefly, after 21 days of culture, an aliquot (1 mL) of 0.3 M pNPP (dissolved in glycine buffer, pH 10.5) was added to each scaffold and to cells cultured on TCPS. After incubation at 37 °C, the reaction was stopped by the addition of 100 µL 5 M NaOH. Standards of pNPP in concentrations ranging from 0 to 50 mM were freshly prepared from dilutions of a 500 mM stock solution and incubated for 10 min with 7 U of ALP (Sigma-Aldrich) previously dissolved in 500 µL of ddH_2_O. The absorbance reading was performed at 405 nm with a microplate reader (BioRad Laboratories, Marnes-La-Coquette, France) using 100 µL of standard or sample placed into individual wells of a 96-well plate. Samples were run in triplicate and compared against a calibration curve of p-nitrophenol standards. The enzyme activity was expressed as millimole of p-nitrophenol produced per minute per milligram of enzyme.

### 2.10. Calcium–Cresolphthalein Complexone Method

To evaluate calcium deposition, the calcium–cresolphthalein complexone method was performed on hFF-MSCs cultured on scaffolds and on controls for 21 days in a culture medium with or without osteogenic factors, as previously described [23]. Briefly, the calcium content of each sample was assayed to quantify the amount of mineralized matrix present, using a Calcium Fast kit (Mercury SPA, Naples, Italy) according to the manufacturer’s instructions. Samples were run in triplicate and compared with the calibration curve of standards.

### 2.11. Gene Expression Analysis

At the end of the culture period (7 and 21 days) in osteogenic medium, total RNA was extracted from hFF-MSCs seeded on TCPS, on titanium and on Ti-bioglass using Trizol^®^ reagent according to the manufacturer’s instructions (Invitrogen, Carlsbad, CA, USA). The RNA from samples was then stored at −80 °C until use. First-strand cDNA was synthesized using an iScript kit (BioRad Laboratories). Real-time qPCR was performed on cDNA, using Quantitative Real-Time PCR (Mini-Opticon^®^ Real-Time PCR System, BioRad, Hercules, CA, USA) for a panel of osteogenic genes expressed in a different manner during bone differentiation. Relative gene expression was calculated using the ΔΔCt method. Essentially, ∆∆Ct is the difference between the ∆Ct values of the treated sample (in our case titanium or Ti-bioglass) and the untreated/control sample (in our case TCPS) with GAPDH (glyceraldehyde-3-phosphate dehydrogenase) as a housekeeping gene. All the samples were analysed in triplicate. Primers were designed according to published gene sequences, and they are listed in Table S1.

### 2.12. Extraction of ECM Proteins and Enzyme-Linked Immunosorbent Assay (ELISA)

In order to evaluate the amount of extracellular matrix (ECM) produced after 21 days of culture in osteogenic medium, the scaffolds and controls were washed extensively with sterile PBS to remove the culture medium and then incubated with sterile lysis buffer made of 20 mM Tris-HCl, 4 M GuHCl, 10 mM EDTA and 0.066% (*w*/*v*) sodium dodecyl sulfate (SDS), pH 8.0, and frozen and thawed several times (range 4–6) to allow the cell lysis and ECM disruption. The total protein concentration of samples was then evaluated with a BCA Protein Assay Kit (Pierce Biotechnology, Inc., Rockford, IL, USA). Calibration curves to measure type-I and -III collagens, decorin, osteopontin, osteocalcin, osteonectin, fibronectin and ALP were performed. Microtiter wells were coated with increasing concentrations of each purified protein, from 10 ng to 2 mg, in a coating buffer (50 mM Na_2_CO_3_, pH 9.5) overnight at 4 °C. Control wells were coated with bovine serum albumin (BSA) as a negative control. To measure the ECM amount of each protein by ELISA, microtiter wells were coated, overnight at 4 °C, with 100 µL of the previously extracted ECM (20 µg/mL in coating buffer). After three washes with PBS containing 0.1% (*v*/*v*) Tween 20, the wells were blocked by incubating with 200 µL of PBS containing 2% (*w*/*v*) BSA for 2 h at 25 °C. The wells were subsequently incubated for 1.5 h at 25 °C with 100 µL of the anti-type-I and -III collagens, anti-osteopontin, anti-osteocalcin, anti-osteonectin and anti-ALP rabbit polyclonal antisera (1:500 dilution in 1% BSA), kindly provided by L. Fisher [11]. The same dilution was used for the anti-fibronectin rabbit polyclonal IgG. After washing, the wells were incubated for 1 h at 25 °C with 100 µL of horseradish peroxidase (HRP)-conjugated goat anti-rabbit IgG (1:1000 dilution in 1% BSA). The wells were finally incubated with 100 µL of the development solution (phosphate–citrate buffer with o-phenylenediamine dihydrochloride substrate). The colour reaction was stopped with 100 µL of 0.5 M H_2_SO_4_, and the absorbance values were measured at 490 nm with a microplate reader (BioRad Laboratories). An underestimation of the absolute protein deposition is possible because the sample buffer, used for matrix extraction, contained sodium dodecyl sulfate, which may interfere with the protein adsorption during ELISA.

### 2.13. Statistical Analysis

Three independent experiments (*N*) (unless otherwise indicated) were performed to generate a statistically significant number of events and to test the reproducibility of the results per each type of experiment from 2 to 3 scaffolds (*n*) (as indicated in the figure legends). The results were expressed as mean ± standard deviation (SD). All statistical comparisons between the two types of scaffolds and the controls were performed using the Kolmogorov–Smirnov method to test data normality, and then one-way analysis of variance followed by Bonferroni’s post hoc test, electing a significance level of 95% (*p* ˂ 0.05).

## 3. Results

### 3.1. Chemical and Morphological Characterization of the Scaffolds

**Figure 1:**
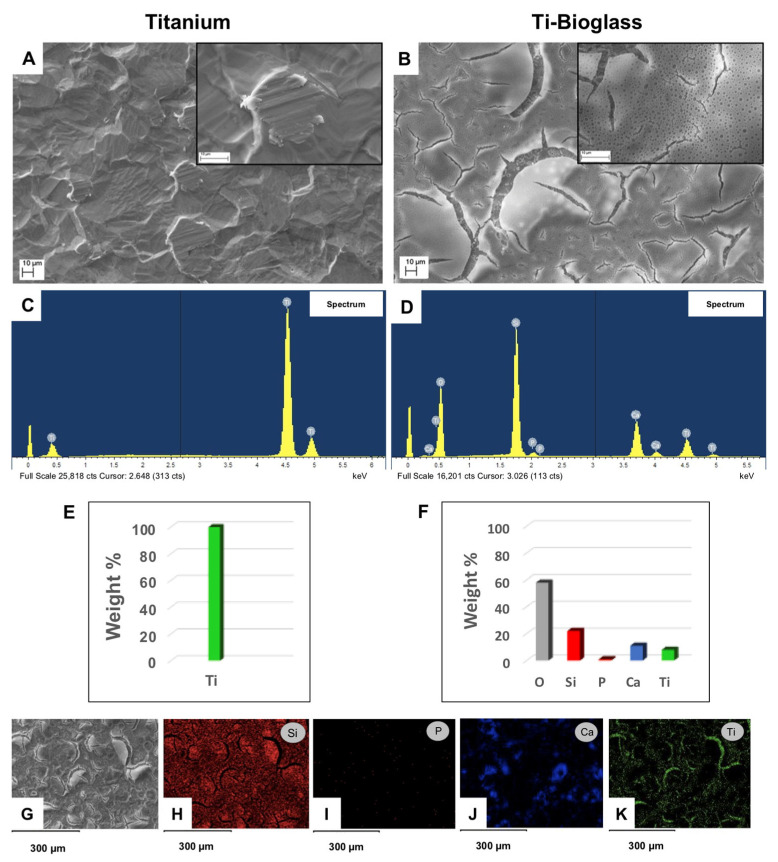
Material surface characterization. Scanning electron microscopy (SEM) images of titanium (**A**) and Ti-bioglass (**B**) disks at magnification 1000× and 5000× (inserts); scale bars represent 10 µm. EDX spectrum for titanium (**C**) and Ti-bioglass (**D**). The relative weight % of the elements detected in both materials in (**E**,**F**), respectively. EDX elemental mapping of Si, P, Ca and Ti elements on Ti-bioglass disk surface (**G**–**K**) relative to the SEM image (**E**) (*n* = 3).

The first phase of our study involved the preparation of 58S bioglass for use as a coating on 99.6% pure titanium scaffolds. Morphological analysis to characterize the surface of both titanium scaffold and titanium scaffold covered with bioglass film was performed by SEM and EDS investigation. Figure 1 shows two micrographs at higher magnification of the surface of pure titanium scaffold (Figure 1A) and titanium scaffold coated by 58S bioglass film (Figure 1B), respectively. EDX analysis of the composition of titanium scaffolds with or without 58S bioglass film coating is indicated in Figure 1C,D, respectively. Figure 1E,F reports the relative weight % of the elements detected in both materials, respectively. As shown in Figure 1D, in addition to the presence of Ti and O, the presence of the characteristic elements of a bioglass such as Si, Ca, and P in the bioglass scaffold was also detected. Observing the elemental distribution map, the bioglass film does not appear to uniformly cover the scaffold surface, with pores having a diameter of the order of 1 µm (Figure 1G–K). The porous part is made of bioglass, characterized by the presence of Si (Figure 1H), P (Figure 1I) and Ca (Figure 1J), while the Ti distribution (Figure 1K) is visible above all where the film is less thick (some superficial cracks are found, possibly formed during the treatment of thermal stabilization of the two materials, which have a different coefficient of thermal expansion).

### 3.2. hFF-MSC Cell Adhesion and Viability

**Figure 2:**
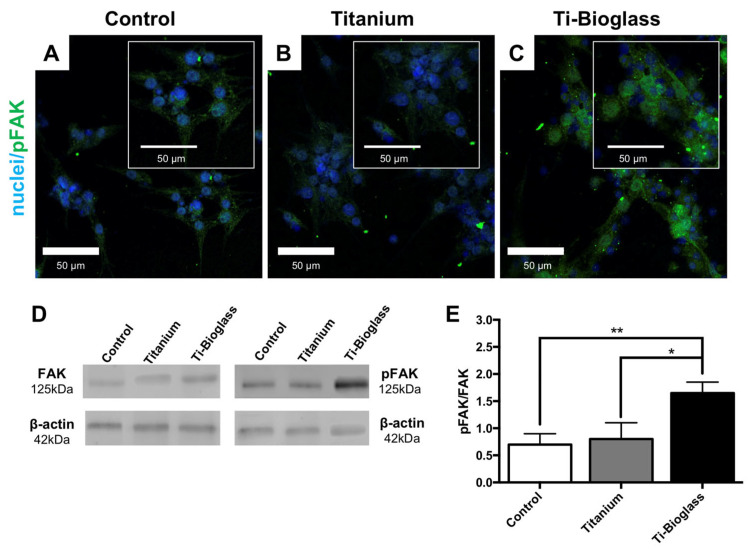
Cell adhesion. Representative CLSM images of phosphorylated focal adhesion kinase (pFAK, green, 488 Alexa Fluor) immunostaining of hFF-MSCs cultured for 24 h on glass coverslip (control (**A**)), titanium (**B**) and Ti-bioglass (**C**) disks. Magnification 40× and 60× (inserts); scale bars represent 50 µm. Nuclei were stained with Hoechst 33342 (blue). Quantitative evaluation by Western blot of FAK and pFAK (**D**) and relative bands densitometric analysis (**E**). Statistical significance values are indicated as ** *p* < 0.01 and * *p* < 0.05; *N* = 3, *n* = 2.

To quantitatively assess cell adhesion, hFF-MSCs seeded on titanium, Ti-bioglass and the control (coverslip or TCPS) were incubated for 24 h and focal adhesion kinase (FAK) activation was assessed by CLSM and Western blot (Figure 2). Interestingly, the hFF-MSCs on the Ti-bioglass scaffold showed more intense green fluorescence immunostaining for phosphorylated FAK (pFAK) than the other cells seeded on coverslips or titanium scaffolds (Figure 2A–C). These data were confirmed by quantitative immunoblot analysis (Figure 2D,E). FAK activation (pFAK signal) was significantly higher in hFF-MSCs grown on Ti-bioglass compared to those on titanium (* *p* < 0.05) and the control (** *p* < 0.01) (Figure 2D,E).

**Figure 3:**
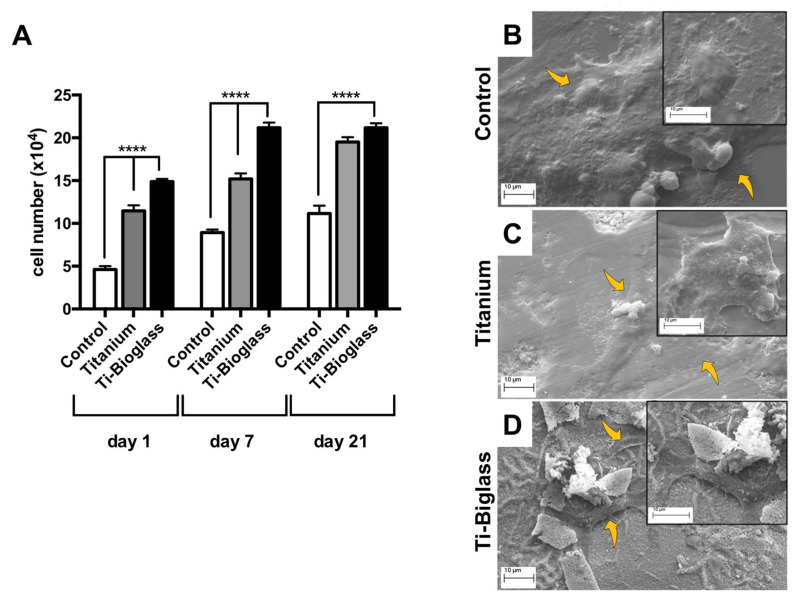
Cell viability. (**A**) Viability of hFF-MSCs cultured on tissue culture plate (control), titanium and Ti-bioglass disks evaluated at 1, 7 and 21 days of incubation. Results are expressed as the number of cells ± SD (standard deviation) (**** *p* < 0.0001, *N* = 3, *n* = 2). Representative SEM images of hFF-MSCs cultured for 21 days on glass coverslip (control (**B**)), titanium (**C**) and Ti-bioglass scaffolds (**D**) at 3000× and 5000× (insert) magnification (scale bars represent 10 µm). All SEM analyses were conducted with an accelerating voltage of 20 kV and high vacuum mode. The yellow arrows indicate the adhered cells on all tested surfaces.

Cell proliferation during the culture period was determined on days 1 and 7 and at the end of the culture period (21 days) (Figure 3A). Overall, a higher number of cells was observed on titanium and Ti-bioglass compared to the control (**** *p* < 0.0001). A significant difference in the number of cells was observed between the titanium and Ti-bioglass at days 1 and 7 (**** *p* < 0.0001), except for day 21, where a slight increase was observed on the Ti-bioglass compared to the titanium scaffold (*p* > 0.05) (Figure 3A). Figure 3B–D contains the SEM micrographs of 21 days of cell culture. SEM images displayed a widespread cell monolayer over the control (Figure 3B). A more adherent cell of fibroblastic shape, with a large central portion containing the nucleus and cytoplasmic protrusions with flattened spindle-shaped ends, covering the titanium (Figure 3C) and Ti-bioglass surfaces (Figure 3D) was observed. Interestingly, cells grown on the Ti-bioglass disks interacted with the bioglass film, insinuating themselves within the surface cracks (Figure 3D). To further characterize the cells derived from the follicular fluid as mesenchymal stem cells, the cell expression of vimentin was also assessed on both 3D scaffolds, after 21 days of culture, in comparison to the cell samples on coverslips. As shown in Figure S2, morphological analysis revealed that all cells adhered to both scaffolds and expressed the same intense cytoplasmic positivity for vimentin (green fluorescence), a cytoskeleton protein specific to mesoderm-derived cells and a characteristic marker of mesenchymal cells, as demonstrated by the quantification of fluorescence intensity of vimentin (Figure S2D).

### 3.3. hFF-MSC Osteogenic Differentiation

**Figure 4:**
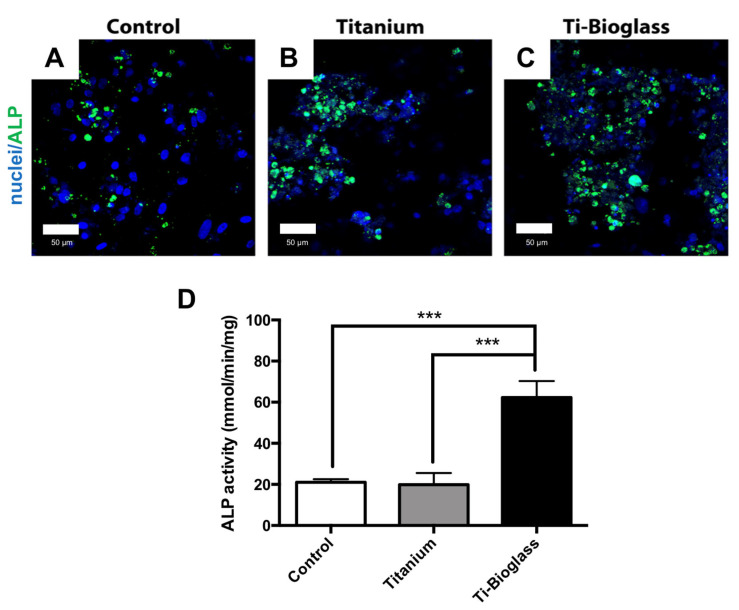
ALP immunolocalization and activity. ALP immunolocalization and activity of hFL-MSCs cultured for 21 days on glass coverslip (control (**A**)), titanium (**B**) and Ti-bioglass (**C**) disks in osteogenic medium. Representative CLSM images of ALP (green, 488 Alexa Fluor) immunostaining of hFL-MSCs. Magnification 40×; scale bars represent 50 µm (**A**–**C**). Nuclei were stained with Hoechst 33342 (blue). ALP-specific activity (**D**) evaluated by enzymatic assay at the end of the culture period as described in the Section 2 in the osteogenic condition. Bars represent the mean ± SD of three experiments (*** *p* < 0.001, *N* = 3, *n* = 2).

To assess the hFF-MSC osteogenic differentiation on the Ti-bioglass disk, all samples (control, titanium and Ti-bioglass) were analysed for bone gene expression, ALP activity and calcified extracellular matrix deposition. Figure 4 and Figure S3 show the ALP activity determined on all samples at the end of the culture period, expressed in mmol/minute/mg protein content. It is worth noting that in hFF-MSCs cultured in the absence of osteogenic factors for 21 days, significantly lower ALP activity values were found than in those cultured in the presence of osteogenic supplements (Figure 4 and Figure S3). Moreover, in a non-osteogenic medium, a non-significant increase in ALP activity was detected in hFF-MSCs on the Ti-bioglass compared to that observed in those on the titanium and control (Figure S3). By contrast, the level of ALP activity was consistently higher on the Ti-bioglass disc than on titanium and the control when osteogenic supplements were added in the culture media (*** *p* < 0.001) (Figure 4D), in agreement with the ALP immunostaining results (Figure 4A–C). 

**Figure 5:**
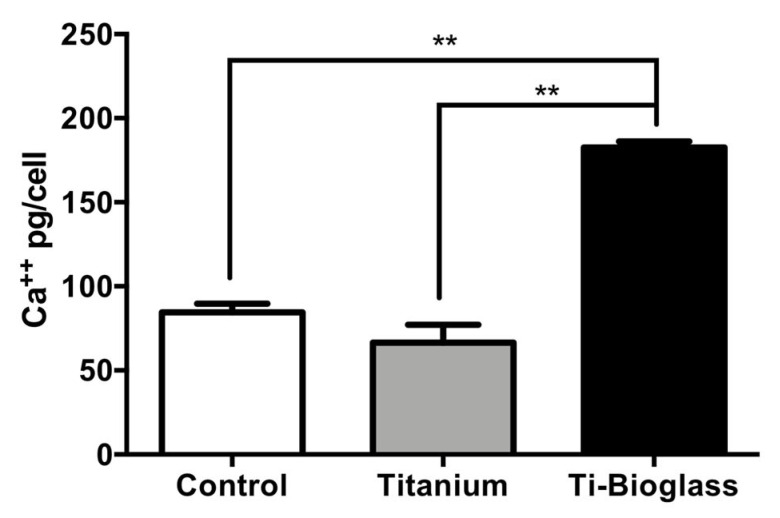
Extracellular calcium deposition. Quantitative evaluation of calcium deposited by hFF-MSCs after 21 days of culture on tissue culture plate (control), titanium and Ti-bioglass disks in osteogenic medium. Results are expressed as pg Ca^2+^/cell and presented as mean ± SD (** *p* < 0.01, *N* = 3, *n* = 2).

Furthermore, at the end of the culture, the calcium–cresolphthalein complex protocol was applied to determine the amount of calcium matrix deposition on all samples, with or without osteogenic factors (Figure 5 and Figure S4). As shown in Figure 5, Ti-bioglass supported an increased calcium deposition by hFF-MSCs compared to titanium and the control when cultured with osteogenic medium (** *p* < 0.01), while a slight increase was detected in the absence of osteogenic factors (Figure S4).

**Figure 6:**
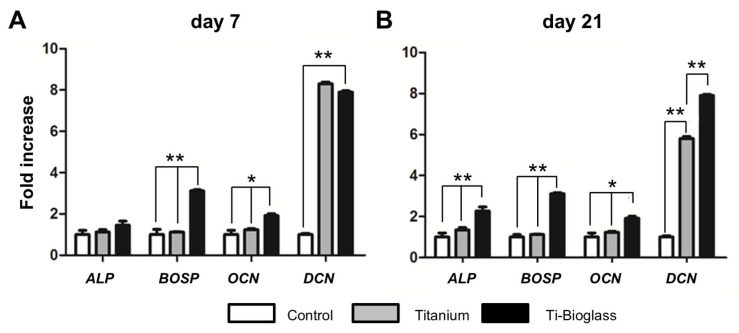
Gene expression of the indicated bone-specific markers as determined by qRT-PCR. hFF-MSCs were cultured for 7 (**A**) and 21 (**B**) days on glass coverslip (control), titanium and Ti-bioglass disks in an osteogenic medium. Bars represent the mean ± SD of three different experiments. Statistical analysis performed against the control and titanium (* *p* < 0.05, ** *p* < 0.01, *N* = 3, *n* = 2).

For these reasons, the subsequent experiments were performed by adding osteogenic factors to the culture media. The gene expression results of bone-specific markers (ALP, BOSP, OCN and DCN) are shown in Figure 6. At 7 days of culture (Figure 6A), the expression of the gene that encodes for ALP was very similar between control, titanium and titanium–bioglass disks, without any statistical differences. By contrast, the BOSP gene was 2.5-fold higher in Ti-bioglass with respect to the other samples (*p* = 0.001), OCN gene 0.5-fold higher (* *p* < 0.05) and DCN 8-fold higher in Ti-bioglass and titanium disks with respect to controls (*p* = 0.001). At 21 days (Figure 6B), for all the osteogenic genes analysed, the effect of the material is strongly confirmed. In fact, there was a significantly higher expression in Ti-bioglass disks with respect to the titanium and control samples (*p* = 0.001, ALP 0.6-fold higher, BOSP 2-fold higher, OCN 1-fold higher and DCN 6-fold higher with respect to control cells and 2-fold higher with respect titanium disks). From these results, it seems that Ti-bioglass at the gene level actively promotes the bone formation of hFF-MSC cells.

To further characterize the osteogenic process and the amount of extracellular matrix constituents produced throughout all samples, an ELISA assay of the extracted extracellular matrix was performed (Table 1). A significant enhancement in bone protein production was observed in Ti-bioglass with increments of 1.63- (OCN), 1.49- (ON), 1.50- (OPN), 1.56- (COL-I) and 1.55-fold (COL-III) in comparison with titanium disk (* *p* < 0.05). By contrast, no significant differences were observed in the expression levels of ALP and FN between the titanium and Ti-bioglass cell cultures (*p* > 0.05).

## 4. Discussion

In this study, human ovarian follicular fluids were used as a new source of mesenchymal stem cells for tissue engineering experimental systems in vitro. The advantage of using this source is that follicular liquid is a human waste material, collected routinely from women undergoing in vitro fertilization treatment and usually discarded after oocyte isolation [15]. This liquid could be collected in large volumes containing up to millions of MSCs with no impact on the effectiveness of IVF, and would represent a significant alternative source of heterogeneous cell populations with epithelial-like, fibroblast-like and neural-like morphologies [15,16,22,26]. This human follicular liquid, as we have demonstrated previously, also contains mesenchymal stem cells which exhibit the expression of some specific markers (CD44, CD73, CD90 and CD105 antigens) which are able to differentiate into osteoblasts, chondrocytes and adipocytes [15,16]. It has also been demonstrated that these FF-derived MSCs have a high level of potential to differentiate into oocyte-like cells (OLC) and that this can be increased by the induction of bone morphogenetic protein 15 (BMP15) [18]. These cells, as indicated in other studies, show a very high degree of plasticity when compared with other MSCs. They exhibit several mesodermal-germ-layer-specific markers and this is indicative of their capacity not only to differentiate into mesodermal tissues but also to form neural-like spheroidal structures [16]. We may conclude, then, that human ovarian follicular fluid constitutes an as yet unutilized source of stem cell materials and, in particular, of stem cells which could be useful for in vitro experimental research models in regenerative medicine. Other studies have shown that this subpopulation of cells, collected from mature ovarian follicles and grown on a 3D-matrix made of type I collagen [26] and on gelatin cryogel scaffolds [20], are able to maintain their morphological and functional characteristics over prolonged time periods, in comparison with MSCs derived from human bone marrow. In this study, we hypothesized an interesting combination of FF-derived MSCs and bioactive glass to induce bone maturation as a novel approach for bone tissue engineering applications. It has been widely shown that the response of cells to materials is closely influenced by surface properties. The interaction between stem cells and materials is critical in regulating differentiation pathways in the early stages [27]. In the case of bioactive glass materials, they interact with several scaffolds through adhesion sites, including integrin receptors [28], which trigger a cascade of intracellular cell signalling molecules that finally, through Cbfa1 transcription factors, activate or deactivate gene expression in osteoblastic cells [29,30]. There exists ample evidence for the osteogenic differentiation of stem cells derived from other tissues, such as adipose tissue [31] and bone marrow [32], when cultured on bioglass-coated materials, and a number of demonstrations of the successful growth and osteogenic differentiation of hFF-MSCs on biomaterials such as chitosan/polyprolactone/zinc scaffolds [33]. Here, for the first time, we have successfully isolated hMSCs from hFF and have observed that a thin coating of bioglass on a 3D Ti scaffold creates a favourable environment for adhesion, proliferation and osteoblastic differentiation. It is significant that the cells adhered to the scaffold and remained viable for at least three weeks, although the proliferative activity showed that cells on Ti-bioglass grew better than on other scaffolds, in line with the ability of MSCs to better migrate on rough surfaces, extruding cytoplasmic protrusions. The qualitative immunofluorescence analysis and quantitative immunoblotting of the focal adhesion kinase FAK, a key downstream component of integrin-mediated signalling which is directly involved in integrin-mediated responses such as cell motility, adhesion, proliferation and differentiation, are also of interest [34]. The hFF-MSCs exhibited the highest immunofluorescence signals when cultured on Ti-bioglass compared to the control and titanium. Furthermore, immunoblotting data quantitatively revealed increased FAK activity in cells seeded on Ti-bioglass. This suggests that this surface was more suitable for early cell/biomaterial interaction. The direct addition into media of osteogenic supplements such as dexamethasone, β-glycerophosphate and ascorbic acid is a common approach to promoting osteogenic cell differentiation for tissue engineering purposes [35]. The osteogenic factors appear to be crucial for the osteogenic commitment of hFF-MSCs on Ti-bioglass scaffolds and the addition of osteogenic factors to the culture medium resulted in significantly increased ALP enzyme activity and calcium deposition in hFF-MSCs cultured in control, titanium and Ti-bioglass compared to cells cultured without. A significant difference in these two important osteogenic markers was also found between titanium and Ti-bioglass in the osteogenic conditions. This is supported by our previous work which demonstrated the crucial role of the osteogenic medium in promoting increased osteogenic differentiation in different scaffold types [25,36,37]. When cultured in a medium containing osteogenic factors, the hFF-MSCs seeded on Ti-bioglass 3D scaffolds showed an up-regulation of early (BOSP and ALP) and late bone-related genes (OCN and DCN) with respect to the other samples. This point is associated with higher production of proteins involved in the bone mineralization process: ON (a glycoprotein that facilitates mineralization and promotes mineral crystal formation), ALP (the major regulator of bone mineralization) and OCN (among the most abundant proteins in mature osteoblast) [38,39]. All these findings support the hypothesis that these stem cells, as with those isolated from other tissues, represent an interesting cellular source for creating three-dimensional bone constructs as experimental tissue models.

## 5. Conclusions

The ability of hFF-MSCs to grow on a 3D scaffold, as has been demonstrated, suggests that these cells are a viable model for regenerative medicine and clinical predictive applications. The in vitro maturation (IVM) of human oocytes remains an important possibility to obtain metaphase II oocytes from pre-antral follicles in pre-pubescent patients or in women who are unable to undergo controlled hyperstimulation with gonadotropins [40,41]. Further, the use of hFF-MSCs as a feeder layer in 3D ovarian constructs may actually improve oocyte competence, during IVM, due to their ability to grow on various scaffolds whilst maintaining some of their original characteristics [38,42,43,44]. In conclusion, in this study we have established that stem cells from ovarian follicular fluid, usually discarded after oocyte retrieval, can adhere to and grow stably on bioglass-coated titanium and can activate the key markers of bone regeneration, such as mesenchymal stem cells derived from other different sources, as reported in the literature [45,46]. This means that hFF-MSCs, in combination with bioglass–titanium transplantation, is a potentially viable new approach to bone and tissue repair and one which, perhaps, may be more adapted to patient-specific applications: in the case of bone tissue engineering, for example, the differentiated cells described in this study could be used to build a new biocompatible surface which is rich in the human bone extracellular matrix.

## Figures and Tables

**Table 1:** Bone matrix protein deposition. Normalized amount of bone matrix proteins secreted and deposited by hFF-MSCs cultured for 21 days on tissue culture plate (control), titanium and Ti-bioglass disks in osteogenic conditions evaluated by ELISA. The results are expressed as pg/cell. Statistical analysis: * *p* < 0.05, ** *p* < 0.01, *** *p* < 0.001 and **** *p* < 0.0001.

	*Control* *(pg/Cell)*	*Titanium* *(pg/Cell)*	*Ratio Related to Control*	*Ti-Bioglass* *(pg/Cell)*	*Ratio Related to Control*	*Ratio* *Ti-Bioglass/* *Titanium*
***ALP***	20.60 ± 3.28	29.82 ± 1.88	1.45 *	45.70 ± 19.97	2.22 ***	1.53
***FN***	1.25 ± 0.19	2.42 ± 1.00	1.93 **	2.96 ± 0.11	2.36 ***	1.22
***OCN***	3.19 ± 0.02	8.18 ± 0.08	2.56 **	13.40 ± 1.14	4.20 ***	1.63 **
***ON***	2.15 ± 001	4.30 ± 0.11	2.00 **	6.40 ± 0.08	2.98 ***	1.49 ****
***OPN***	36.95 ± 2.64	47.71 ± 1.37	1.29	71.81 ± 3.02	1.95 **	1.50 ***
***COL-I***	100.40 ± 1.46	155.07 ± 1.24	1.54 *	242.13 ± 27.35	2.41 ***	1.56 **
***COL-III***	84.50 ± 3.36	113.74 ± 3.79	1.35 *	175.31 ± 15.14	2.07 ***	1.54 **

Abbreviations: ALP, alkaline phosphatase; FN, fibronectin, OCN, osteocalcin; ON, osteonectin; OPN, osteopontin; COL-I, type-I collagen; COL-III, type-III collagen.

## Data Availability

The data presented in this study are available on request from the corresponding authors.

## Supplementary Materials

The following supporting information can be downloaded at: https://www.mdpi.com/article/10.3390/ma16103676/s1, Figure S1: Assessment of mesenchymal properties of hFF-MSCs; Figure S2: Vimentin immunofluorescence; Figure S3: ALP immunolocalization and activity in the absence of osteogenic factors; Figure S4: Extracellular calcium deposition in the absence of osteogenic factors; Table S1: List of primers used.
