# Supplementary material for: Human Ovarian Follicular Fluid Mesenchymal Stem Cells Express Osteogenic Markers When Cultured on Bioglass 58S-Coated Titanium Scaffolds
Source: Materials (Basel). 2023 May 11;16(10):3676. doi: 10.3390/ma16103676 (PMC10222050; doi:10.3390/ma16103676)
Supplement: Supplementary file 1 [file materials-16-03676-s001.zip › materials-2345297-supplementary.pdf]

Supplementary Material

Human ovarian follicular fluid Mesenchymal Stem Cells express osteogenic markers when cultured on Bioglass 58S-coated Titanium scaffolds

Federica Riva[1,†], Nora Bloise[2,3,†], Claudia Omes[4,*], Gabriele Ceccarelli[5], Lorenzo Fassina[6], Rossella Elena Nappi[7,8], Livia Visai[2,3,*]

[1]Histology and Embryology Unit, Department of Public Health, Experimental and Forensic Medicine, 27100 University of Pavia, Italy

[2]Department of Molecular Medicine, Centre for Health Technologies (CHT), INSTM UdR of Pavia, University of Pavia, 27100 Pavia, Italy

[3]Medicina Clinica-Specialistica, UOR5 Laboratorio di Nanotecnologie, ICS Maugeri, IRCCS, 27100 Pavia, Italy

[4]Center for Reproductive Medicine, Obstetrics and Gynecology Unit 2, Woman and Child Health Department, Fondazione IRCCS Policlinico San Matteo, 27100 Pavia, Italy

[5]Human Anatomy Unit, Department of Public Health, Experimental and Forensic Medicine, Centre for Health Technologies (CHT), University of Pavia, 27100 Pavia, Italy

[6]Department of Electrical, Computer and Biomedical Engineering, Centre for Health Technologies (CHT), University of Pavia, 27100 Pavia, Italy

[7]Department of Clinical, Surgical, Diagnostic and Pediatric Sciences, University of Pavia, 27100 Pavia, Italy

[8]Center for Reproductive Medicine, Obstetrics and Gynecology Unit 2, Woman and Child Health Department, Fondazione IRCCS Policlinico San Matteo, 27100 Pavia, Italy

†These authors have contributed equally to this work

*** Corresponding Authors**
livia.visai@unipv.it

c.omes@smatteo.pv.it

Table S1

List of primers used.

Gene	FW	RW
**ALP**	5′ CTA TCC TGG CTC CGT GTC C 3′	5′ AGC CCA GAG ATG CAA TCG 3′
**BOSP**	5′ GGG CAG TAG TGA CTC ATC CG 3′	5′ TCA GCC TCA GAG TCT TCA TCT TC 3′
**OCN**	5′-CTT CAG ACT GCC CGG AGA-3′	5′ GAA AGA AGA TCC AGG CCC TC-3′
**DCN**	5′ ACC CCC TCC TCC TTT CCA CAC C 3′	5′ ACC AGG GAA CCT TTT AAT CCG GGA A 3′
**GAPDH**	5′ AGC CTC AAG ATC ATC AGC AAT GCC 3′	5′ TGT GGT CAT GAG TCC TTC CAC GAT 3′
**Abbreviations:** ALP, alkaline phosphatase; BOSP, bone sialoprotein; OCN, osteocalcin; DCN, decorin, GAPDH, glyceraldehyde phosphate dehydrogenase		

Figure S1

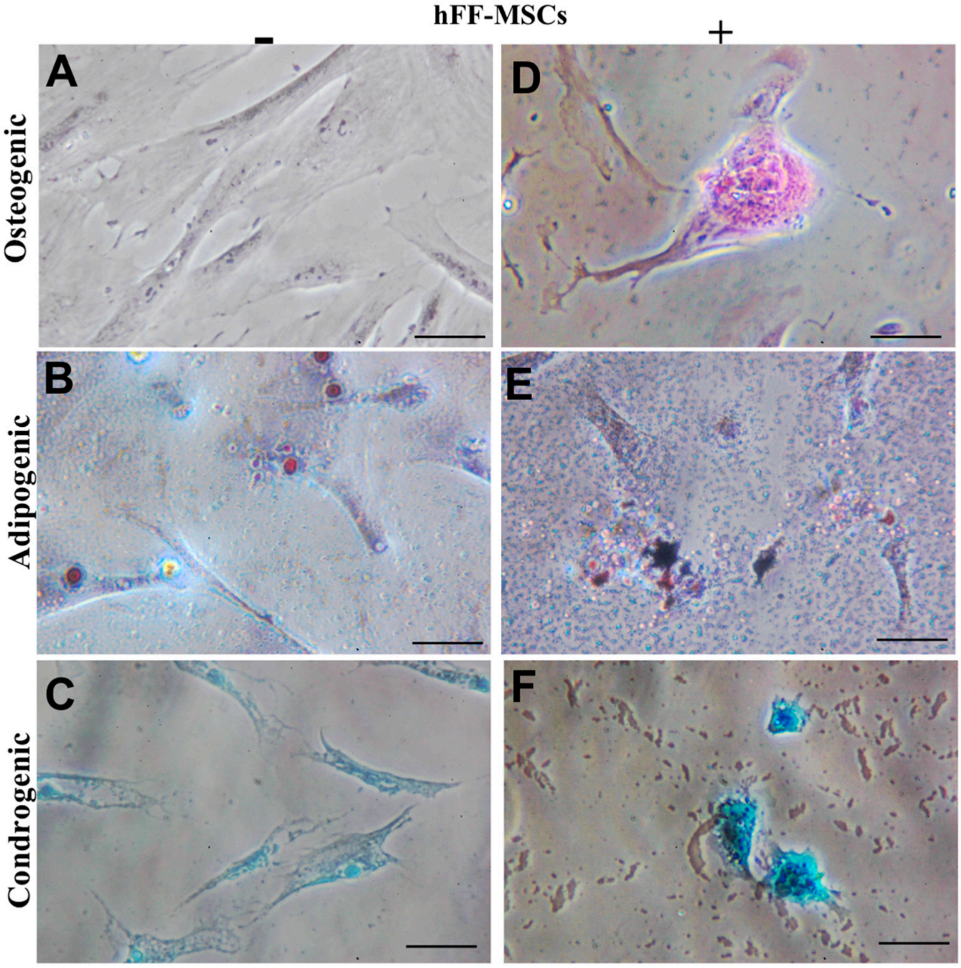

Figure S1 Assessment of mesenchymal properties of hFF-MSCs. Isolated stromal mesenchymal stem cell from ovarian follicular fluid FF-MSCs at [p1] culture passage, grown in basal medium (A,B,C) and in osteogenic (D), adipogenic (E) and chondrogenic (F) differentiating conditions. Assessment of calcium accumulation was visualized by Von Kossa staining (A,D) as mineralized area around the cells. O-Red Oil staining shows intracytoplasmic lipid drops inside the differentiated cells vs undifferentiated cells (B,E) and Alcian Blue stained the proteoglycans (C,F). All samples were observed with light contrast microscope (Obj Magn. 32×, scale bar 10 μm).

Figure S2

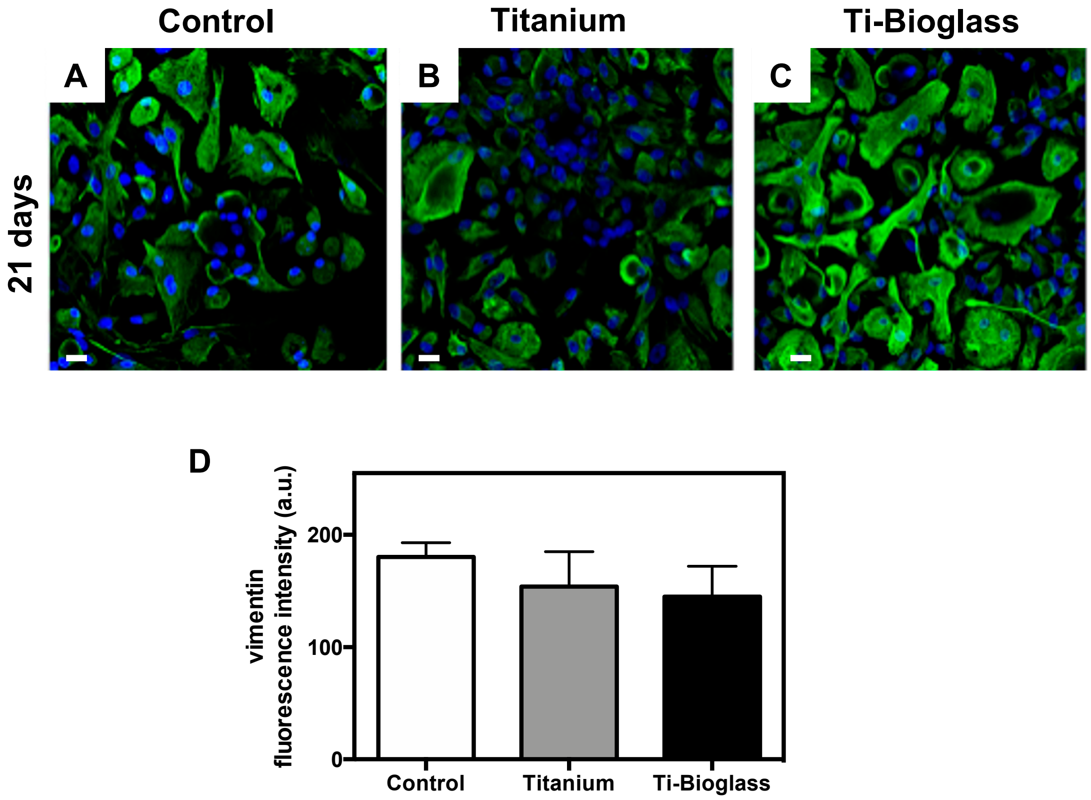

Figure S2 Vimentin immunofluorescence. Representative CLSM images of vimentin immunostaining for cells cultured until 21 days of culture on tissue culture plate (Control, **A**), Titanium (**B**) and Ti-Bioglass (**C**). Magnification 20×, scale bars represent 20 μm. **D**) Quantification of vimentin fluorescence (green) obtain by Image J software. Nuclei were stained with Hoechst 33342 (blue). The results are expressed by arbitrary unit (a.u.) and presented as means ± SD.

Figure S3

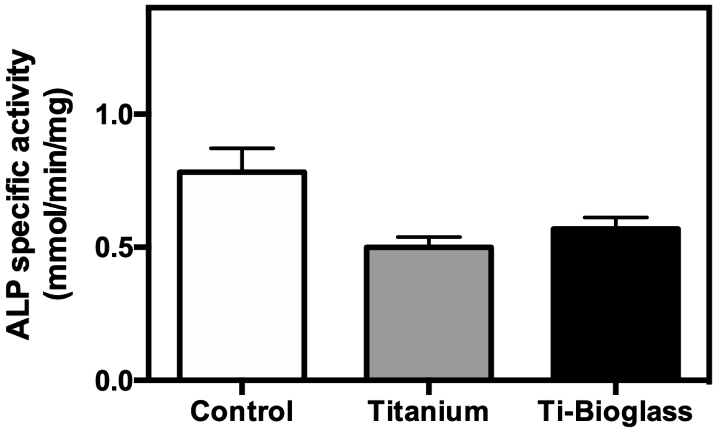

Figure S3 ALP immunolocalization and activity in absence of osteogenic factors. ALP specific activity evaluated by enzymatic assay at the end of culture period as described in Materials and Methods Section 2 in absence of osteogenic medium. Bars represent mean and ± SD of three experiments.

Figure S4

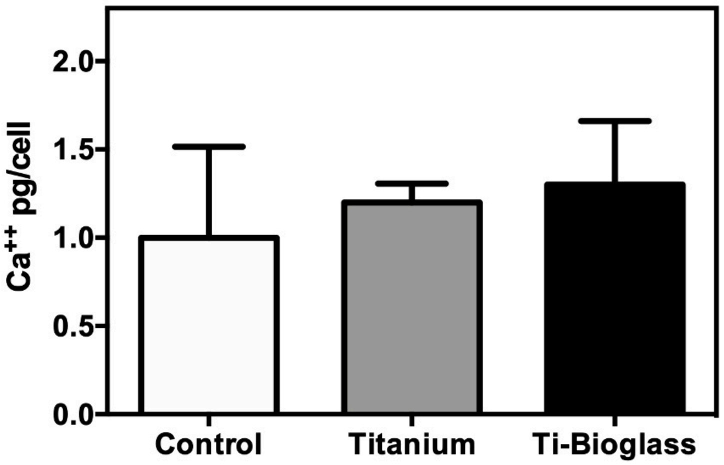

Figure S4 Extracellular calcium deposition in absence of osteogenic factors. Quantitative evaluation of calcium deposited by hFF-MSCs after 21 days of culture on tissue culture plate (Control), Titanium and Ti-Bioglass disks in absence of osteogenic condition. Results are expressed as pg Ca^{2+}/cell and presented as mean ± SD of three experiments.
